# Isolation and molecular identification of endophytic diazotrophs from seeds and stems of three cereal crops

**DOI:** 10.1371/journal.pone.0187383

**Published:** 2017-10-30

**Authors:** Huawei Liu, Lei Zhang, Aihua Meng, Junbiao Zhang, Miaomiao Xie, Yaohong Qin, Dylan Chase Faulk, Baohong Zhang, Shushen Yang, Li Qiu

**Affiliations:** 1 College of Life Sciences, Northwest A&F University, Yangling, Shaanxi, China; 2 College of Veterinary Medicine, Northwest A&F University, Yangling, Shaanxi, China; 3 Department of Biology, East Carolina University, Greenville, North Carolina, United States of America; Dokuz Eylul Universitesi, TURKEY

## Abstract

Ten strains of endophytic diazotroph were isolated and identified from the plants collected from three different agricultural crop species, wheat, rice and maize, using the nitrogen-free selective isolation conditions. The nitrogen-fixing ability of endophytic diazotroph was verified by the *nifH*-PCR assay that showed positive nitrogen fixation ability. These identified strains were classified by 879F-RAPD and 16S rRNA sequence analysis. RAPD analyses revealed that the 10 strains were clustered into seven 879F-RAPD groups, suggesting a clonal origin. 16S rRNA sequencing analyses allowed the assignment of the 10 strains to known groups of nitrogen-fixing bacteria, including organisms from the genera *Paenibacillus*, *Enterobacter*, *Klebsiella* and *Pantoea*. These representative genus are not endophytic diazotrophs in the conventional sense. They may have obtained nitrogen fixation ability through lateral gene transfer, however, the evolutionary forces of lateral gene transfer are not well known. Molecular identification results from 16S rRNA analyses were also confirmed by morphological and biochemical data. The test strains SH6A and MZB showed positive effect on the growth of plants.

## Introduction

Nitrogen is important for the growth of plants and animals [1]. Nitrogen is a staple-basic element of cells, amino acids, DNA, and RNA in living organisms [2]. Under natural conditions, some plant species can build symbiotic relationships with a different range of micro-organisms which are beneficial to each other [3]. Some micro-organisms are able to fix atmosphere nitrogen and provide it to host plants in a process known as biological nitrogen fixation [4]. These micro-organisms are called nitrogen fixing bacteria and they can convert nitrogen into ammonia [5]. Döbereine and Ruschel isolated the first nitrogen-fixing bacteria named *Beijerinckiu flurninensis* from sugarcane in 1958. Shortly afterwards, numerous nitrogen-fixing bacteria were isolated and identified from gramineous plants such as maize, rice, and wheat [6]. In the last two decades, new nitrogen-fixing bacteria were isolated from plant tissues, they were called endophytic diazotrophs and classified as *Azospirillum brasiense*, *Acetobacter diuzotrophicus*, *Azoarcus spp*., *Herbaspirillum seropedicae*, *Herbaspirillum rubrisubalbicans* and *Burkholderia sp*. Endophytic diazotrophs colonize in the internal organization of host plants without causing any harm to the hosts, and they are protected by the endophytic diazotrophs to avoid the interference of external environment and competition [7]. *Acetobacter diuzotrophicus* and *Herbaspirillum seropedicae* were the first certified endophytic nitrogen-fixing bacteria [6, 8]. Endophytic diazotrophs that can fix nitrogen biologically and supply fixed nitrogen to hosts directly, may have a significant role in promoting the sustainable production of crops [9].

The classification of bacteria is based on genotypic [10] and/or phenotypic [11] features. Phenotype can be built on morphological characteristics, physiological property, or biochemical aspects. Genotyping includes lots of methods such as Polymerase Chain Reaction (PCR), rRNA fragmentation analysis, DNA:DNA hybridization, randomly amplified polymorphic DNA (RAPD) [12] fingerprinting, DNA: rRNA hybridization and restriction fragment length polymorphism (RFLP) [13].

Recently, reports describing the isolation and identification of endophytic diazotrophs from the seeds, stems, and leaves of crops have rarely been published. In this study, we isolated and identified endophytic diazotrophs from the seeds and stems of crops in northwest China. Based on RAPD analysis and phylogeny analysis of 16S rRNA, we suggest that these isolates belong to the new endophytic diazotrophs, and some of them had positive effect on the growth of plants.

## Materials and methods

### The collection and preparation of experiment materials

The seeds of wheat (*Triticum aestivum*), maize (*Zea mays*), and rice (*Oryza sativa*) used in this study were collected from Shaanxi province China. The seeds used in this experiment were picked up from the above crops and kept in storage for the use of subsequently. The stems were from two weeks of the seedlings without any cross contamination, and all practices were under aseptic conditions.

### Isolation of endophytic diazotrophs and culture conditions

Seed and stem samples were from rice, maize, and wheat which grow in northwest China. The isolation of endophytic diazotrophs was conducted according to a previous report [14]. Materials were collected and soaked in sterile water for 2 to 3 days, soaking materials were surface sterilized by 75% ethanol for 3 min and washed 3 times with sterile water, and then kept in 0.1% mercuric chloride for 10 min and washed with sterile water 6 times. Sterile treated seeds and stems were cut into slices and inoculated on beef extract-peptone medium agar plates at 28°C for 2 days, and then the bacterial colonies were streaked on three different solid nitrogen-free mediums at 28°C in aerobic conditions until a single colony formed. The three different nitrogen-free media were yeast manitol agar (YMA), yeast extract mannitol (YEM) and tryptone yeast extract (TY). YMA agar medium (pH = 7.0) was composed of 10 g/L mannitol, 0.5 g/L K_2_HPO_4_, 0.2 g/L MgSO_4_, 0.1 g/L NaCl, 3 g/L yeast extract, 3 g/L CaCO_3_, and 15 g/L agar. YEM agar medium (pH = 7.0) included 10g/L mannitol, 0.5 g/L K_2_HPO_4_, 0.2 g/L MgSO_4_, 0.1 g/L NaCl, 1 g/L yeast extract, 15 g/L agar, and 0.025 g/L Congo red. TY (pH = 7.0) had the following composition: 5 g/L tryptone, 3 g/L yeast extract, and 0.66 g/L CaCl_2_. The isolated endophytic diazotrophs were cultivated in TY at 28°C [14] and conserved as glycerin suspension (25%, v/v, in distilled water) at -80°C.

### The extraction of genomic DNA

Genomic DNA of the isolates was prepared based on the method of Cleenwerck [15]. Genomic DNA was dissolved in 50 μL TE buffer solution (pH = 8.0) and maintained at -20°C. TE buffer solution contains 10mM Tris-HCl and 1mM EDTA.

### Amplification of 16S rRNA, nifH and nodA gene fragments

Amplification of the 16S rRNA gene fragment was completely finished by using the universal primer 8F (5′-AGAGTTTGATCCTGGCTCAG-3′) and 1510R (5′-GGCTACCTTGTTACGTA-3′) [16], crude DNA was used for PCR amplification template. The primers Zehrf (5′-TGYGAYCCNAARGCNGA-3′) and Zehrr (5′-ADNGCCATCATYTCNCC-3′) [17] were used for amplification of an about 300-bp segment of the *nifH* gene. The amplification was conducted as described by a previous report [18]. The 50 μL *nifH*-PCR system was as follows: double distilled water 19 μL, primer Zehrf (10mM) 2 μL, primer Zehrr (10 mM) 2 μL, 2×Reaction Mix (TIANGEN BIOTECH (BEIJING) CO. LTD) 25 μL, template DNA 2 μL. The PCR conditions were as follows: preheating (94°C, 5min), 35 cycles of denaturing (94°C, 1min), annealing(50°C, 1min), extension(72°C, 2min), and finally, stable extension for 10min at 72°C. PCR products were stored at 4°C, or resolved in 2.5% agarose gel for electrophoresis analysis. The majority of nitrogen fixing bacteria can not only fix atmospheric nitrogen but also induce the nodule formation in host plants. To find out whether the isolates had the potential to induce nodules, the degenerate primers *nodA*-F (5′-TGCRGTGGAARNTRNNCTGGGAAA-3′) and *nodA*-R (5′-GGNCCGTCRTCRA AWGTCARGTA-3′) were used to amplify the *nodA* gene [19]. The PCR reaction was conducted using the following conditions: preheating (94°C, 5min), followed by 20 cycles of denaturing (94°C, 30s), annealing (60°C to 50°C, 30s) and extension (72°C, 42s), followed by 22 cycles of denaturing (94°C, 30s), annealing (50°C, 30s), extension(72°C, 42s), and final extension (72°C, 7min). The PCR products above were separated using 1% agarose gel; DL2000 was used as a size marker.

### Comparison of 879F-RAPD patterns and 16S rRNA RFLP fingerprinting

The single primer 879F (5′-GCCTGGGGAGTACGGCCGCA-3′) [20] was used for grouping species isolated in this experiment. Crude DNA (2 μL) was used for a PCR amplification template. The RAPD patterns were obtained according to a previous report [20]. PCR conditions were as follows: preheating at 95°C for 5min; 35 cycles of denaturing at 95°C for 1min, annealing at 54°C for 1min, extension at 72°Cfor 2min, and a final extension at 72°C for 7 min. Polymerase chain reaction-restriction fragment polymorphism (PCR-RFLP) of 16S rRNA is a simple way to limit PCR cycles to reduce the possible bias [21]. We used the PCR-RFLP analysis on 16S rRNA genes to study the diversity of the isolates. Two different restriction enzymes (*Hin*fI and *Msp*I) were selected for RFLP analysis. 10 μL of each 16S rRNA PCR products were subjected to restriction enzyme digestion by *Hin*fI and *Msp*I (Takara). Cleavages were conducted overnight at 37°C to guarantee that complete restriction fragments were achieved. RAPD and RFLP results were analyzed in 2.5% agarose gel by following the user instructions and staining with 10000× Andy Gold^™^ Nucleic Acid Gel Stain (Applied BioProbes).

### 16S rRNA gene sequencing and phylogenetic analysis

Based on the results of 879F-RAPD patterns and 16S rRNA RFLP fingerprinting, the desired 16S rRNA gene fragments were purified by using Universal DNA Purification Kit (TIANGEN BIOTEH BEIJNG CO. LTD.) and the purified 16S rRNA amplicons were sequenced by the Shenggong Company in Shanghai, China. The obtained sequences were then compared with those from NCBI (National Center of Biotechnology Information) by using the online BLASTN program [22], and aligned by using the CLUSTAL_W software [23]. Kimura’s two-parameter model [24] was used to calculate the evolutionary distance. The neighbor-joining method [25] was used to generate phylogenetic trees. Bootstrap analysis was based on 1000 resamplings. The MEGA 6 package [26] was used for all above analyses.

### Plant inoculation experiment

The plant inoculation experiment was carried out with three bacteria (SH6B, MZB and GYB) isolated from wheat and rice. These wheat and rice bacteria isolates were detected for their effects on plant growth.

Plants seeds of wheat cv. Xiaoyan 22, were collected and soaked in sterile water for 4h, soaking seeds were surface sterilized by 75% ethanol for 3 min, washed 3 times with sterile water, and then kept in 1% NaClO for 10 min and washed with sterile water for 6 times. Sterilized seeds were put on moist filter papers at room temperature (22±2°C for 2 days in the dark at 28°C to germinate. Germinated seeds were planted and grown in vermiculite at 25°C for 16 h photoperiod with light and at 18°C for 8h in dark.

Three bacteria (SH6B, MZB and GYB) were cultured for 2 days at 28°C in TY liquid medium and then suspended in phosphate-buffered saline (PBS, pH 7.4) to make the OD value of three bacteria between 0.8–1. Then the corresponding experimental groups of wheat seedlings were inoculated with 1mL of bacteria solutions respectively which included SH6B, MZB and GYB by following the method of Diouf et al. [27]. Equal volume (1mL PBS) was added to control groups without any bacteria. Nine biological repeats were performed for each experimental bacteria and control groups. Each test plant was watered by an average of four days with 50 mL half strength of Hoaglang medium.

## Results

### Isolation of endophytic diazotrophs and biochemical analysis

Ten bacteria were isolated from wheat seeds (SH6A, SH6B and ZY1A), maize stems (KHSA), maize seeds (B73A and KHB), and rice seeds (MZA, MZB, GYA and GYB) by using the selective medium YMA and YEM. Gram staining analysis shows that the strains SH6A, SH6B, ZY1A, KHSA, KHB, B73A, MZA, and GYB were of Gram-negative, but the strains MZB and GYA were of Gram-positive; the results complied with the general characteristics of nitrogen-fixing bacteria. The results are shown in Table 1.

**Table 1:** Characteristics of the isolates used in this study.

Name	Isolation materials	Isolation media	Gram’s reaction	Closest relative in database	*nif*H gene amplification
SH6A	Wheat seeds	YMA/YEM	-	*Enterobacter* sp. CZBSD2	+
SH6B	Wheat seeds	YMA/YEM	-	*Klebsiella* sp. Tam9	+
ZY1A	Wheat seeds	YMA/YEM	-	*Klebsiella* sp. Tam9	+
KHSA	Maize seeds	YMA/YEM	-	*Enterobacter* sp. ZYXCA1	+
KHB	Maize stems	YMA/YEM	-	*Pantoea dispersa* strain g42	+
B73A	Maize seeds	YMA/YEM	-	*Enterobacter* sp. TAX9	+
MZA	Rice seeds	YMA/YEM	-	*Pantoea* sp. CT2	+
MZB	Rice seeds	YMA/YEM	+	*Klebsiell*a sp. Tam9	+
GYA	Rice seeds	YMA/YEM	+	*Paenibacillus* polymyxa YRL13	+
GYB	Rice seeds	YMA/YEM	-	*Klebsiella* sp. Tam9	+

Note: + positive, − negative

### Analysis of *nifH* and *nodA* gene fragments

**Fig 1:**
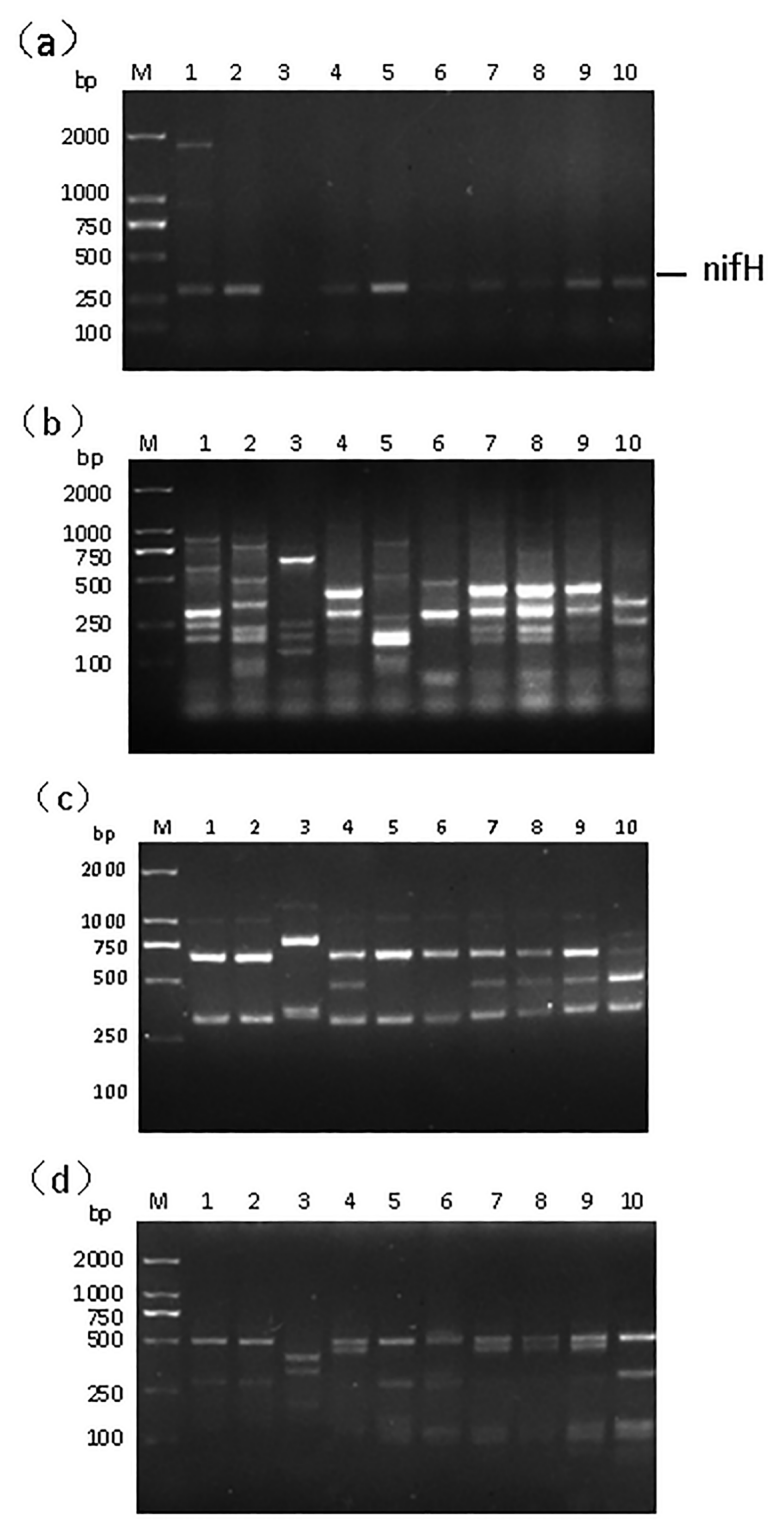
(a) *nif*H gene fragments amplification (b) 879F-RAPD patterns, (c) 16S rRNA *Hin*fI-RFLP fingerprinting, (d) 16S rRNA *Msp*I-RFLP fingerprinting. DL2000 DNA marker (M), SH6A (lane 1), KHSA (lane 2), GYA (lane 3), ZY1A (lane 4), B73A (lane 5), KHB (lane 6), SH6B (lane 7), MZB (lane 8) and GYB (lane 9) and MZA (lane 10).

As nitrogen fixing bacteria, they have a strong or weak ability of nitrogen fixation, at the same time they may also be capable of inducing nodules. The *nifH* gene is very important to biological nitrogen fixation which encodes nitrogen reductase of the nitrogenase, and it can be used as a nitrogen fixing tag. Nodulation (nod) genes which encode Nod factors contain a lot of nodule related genes, among them *nodA* and *nodC* as molecular markers of nitrogen fixing bacteria are important to nodules [28]. In the study, about 300-bp *nifH* gene fragments (Fig 1a) were obtained from the ten isolates, however, there were not *nodA* genes in the ten bacteria.

### Comparison of 879F-RAPD patterns and 16S rRNA RFLP fingerprinting

Compared with other methods for species identification by DNA-based techniques, PCR-RFLP of 16S rRNA offered the greatest sensitivity of species detection [29]. Genotype characterization based on RAPD is easily operating and rapidly advancing. Moreover, in a previous study it was proved that the strains of nitrogen fixing bacteria showing the same RAPD patterns had identical intergenic spacer sequences and 16S rRNA gene and, therefore, RAPD fingerprinting was a preferred tool for classifying the strains with the purpose of selecting typical ones as these phylogenic markers [19]. The result of both 879F-RAPD patterns and 16S rRNA RFLP fingerprintings were shown in Fig 1b, 1c and 1d. Compared with the results from 879F-RAPD patterns and RFLP patterns, we found that the results of two methods had significant differences. The ten isolates were divided into 7 groups by using the 879F-RAPD method. However, they were divided into 5 groups by using the 16S rRNA RFLP method. Obviously, the fingerprint of 879F-RAPD is more elaborated than that of RFLP. The RFLP assay is laboriously and time consuming. On the contrary, the development and application of RAPDmethod is largely makes up for the inadequacy of RFLP method; genotypic identification based on RAPD might replace or supplement RFLP [12]. The results of 879F-RAPD patterns were more reliable and could be used for subsequent analysis of the study.

Based on the results of 879F-RAPD patterns, the strain SH6A presented pattern I. The strain KHSA showed pattern II. The strain GYA presented pattern III. The strains ZY1A, SH6B, MZB and GYB showed pattern IV. The strain B73A presented pattern V. The strain KHB showed pattern VI. The strain MZA presented pattern VII. These results illustrated the species diversity of the isolates from different agricultural plant seeds and stems, and that the isolates were distributed into seven groups from each representative strain, which was chosen for 16S rRNA sequencing.

### 16S rRNA gene sequencing and phylogenetic analysis

**Fig 2:**
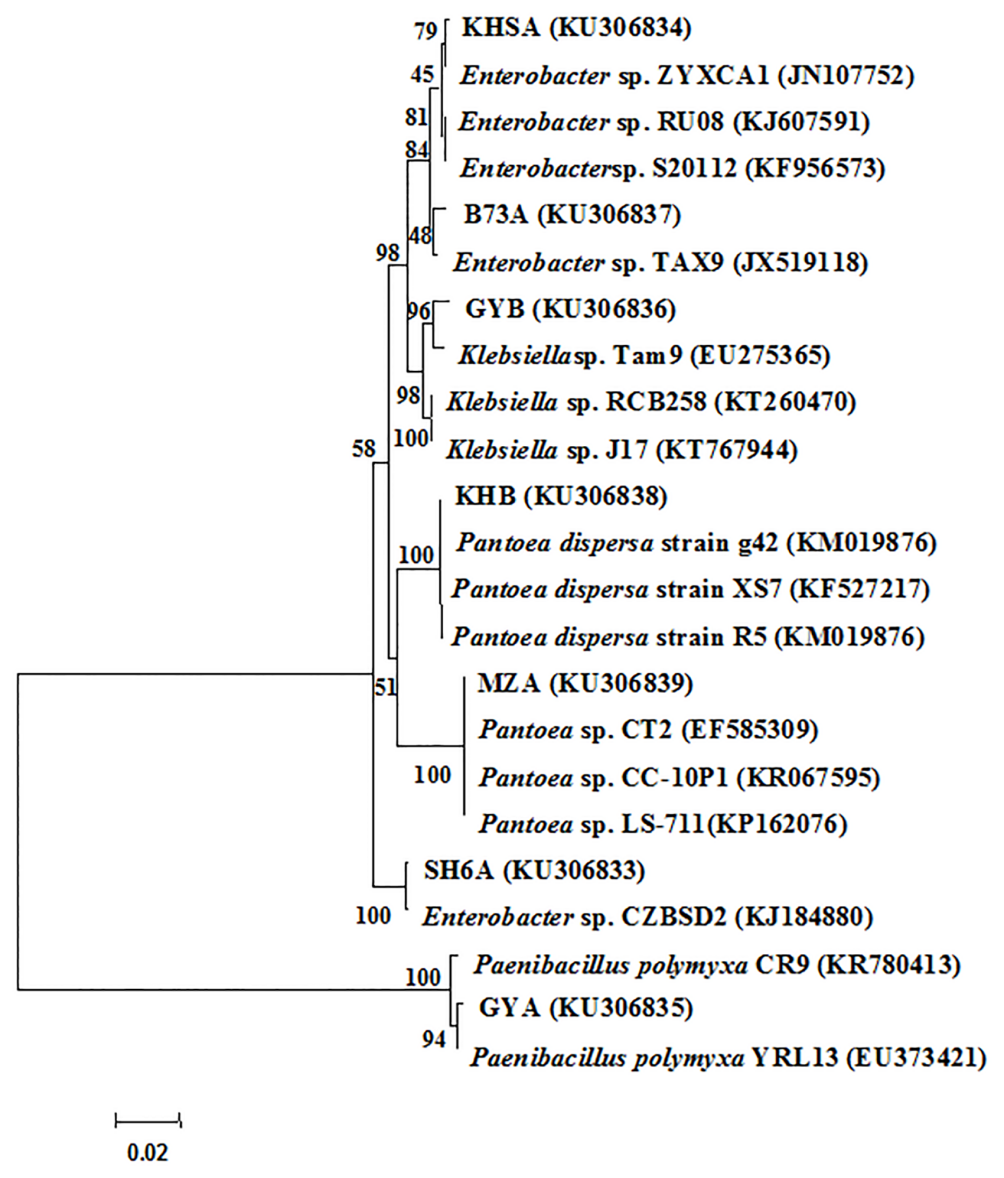
Comparative analysis of 16S rRNA sequence from the RAPD strains SH6A, KHSA, GYA, B73A, KHB, GYB, MZA and representative strains from the GenBank. The significance of each branch is indicated by a bootstrap value calculated for 1000 subsets. Scalebar, 2 nt substitution per 100 nt.

In the current study, classification and identification of nitrogen-fixing bacteria was based on the analysis of their 16S rRNA gene sequences, and therefore, the 16S rRNA sequences from the representative strain of the seven 879F-RAPD groups was obtained and compared with those from NCBI. High sequence homologies were found among the isolated strains and some species of *Enterobacter*, *Klebsiella*, *Pantoea*, and *Paenibacillu* (Fig 2). The strain SH6A represented from RAPD pattern I had a close relation to *Enterobacter* sp. CZBSD2 with 99.79% identity. The strain KHSA represented from the RAPD pattern II had a close relation to *Enterobacter* sp. ZYXCA1 with 99.64% identity. The strain GYA represented from the RAPD pattern III had a close relation to *Paenibacillus polymyxa* YRL13 with 99.72% identity. The strain GYB represented from the RAPD pattern IV had a close relation to *Klebsiella* sp. Tam9 with 99.07% identity. The strain B73A represented from the RAPD pattern V had a close relation to *Enterobacter* sp. TAX9 with 98.86% identity. The strain KHB represented from the RAPD pattern VI had a close relation to *Pantoea dispersa* strain g42 with 100% identity. The strain MZA represented from the RAPD pattern VII had a close relation to *Pantoea* sp. CT2 with 99.86% identity. This promiscuity may confer a significantly information that a broad range of nitrogen fixing bacteria species can inhabit in the same plants.

### The effect of isolated endophytic diazotrophs on plant growth

**Fig 3:**
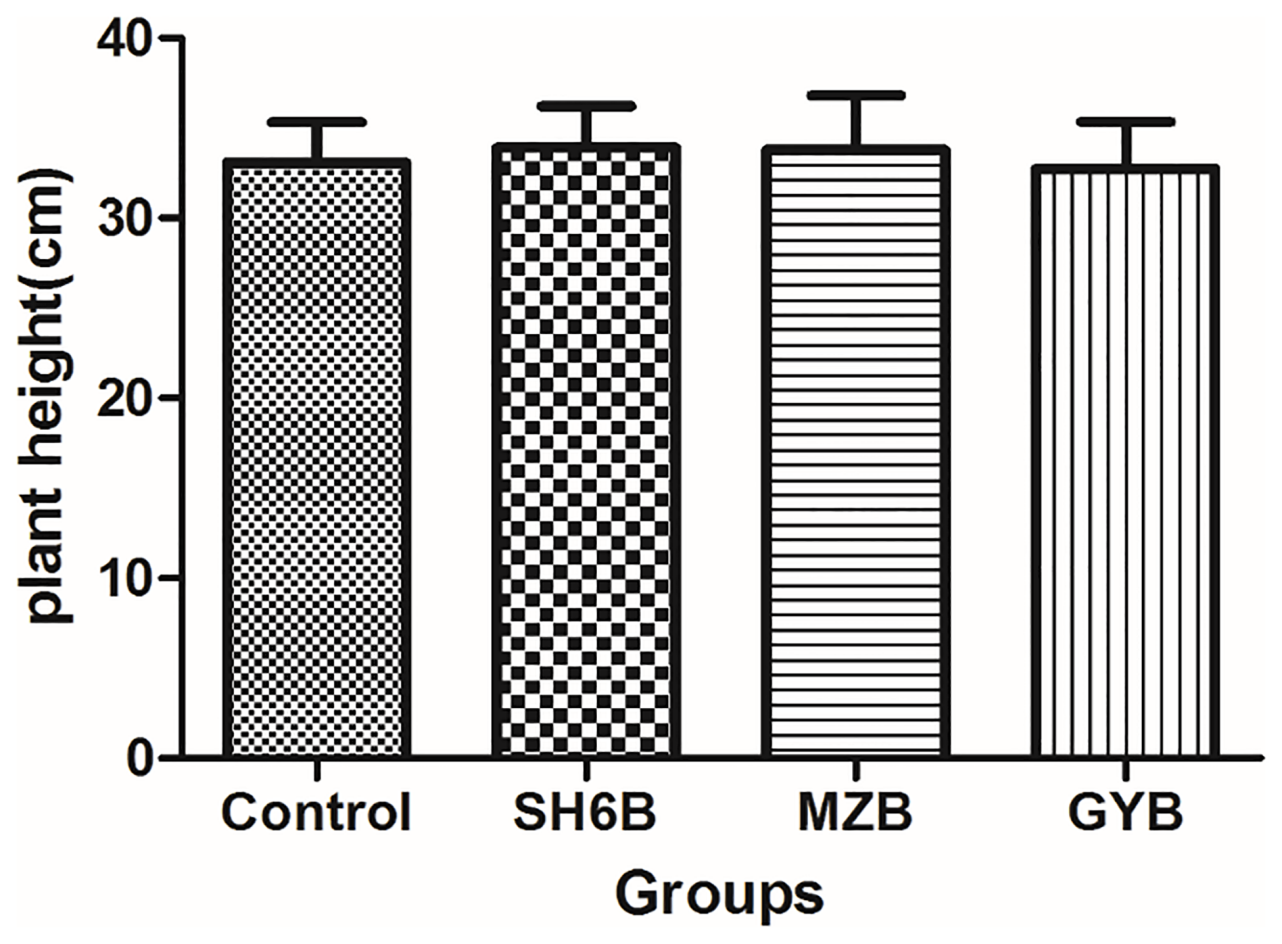
Effect of isolated strains SH6B, MZB and GYB on plant height.

Thirty five days after inoculation, the plant height of each experimental groups and control groups were measured. The comparative results were shown in Fig 3, the strains SH6B and MZB could stimulate slight plant growth, but the strain GYB had little effect on the plant growth. The results revealed that some endogenous nitrogen-fixing bacteria had more or less positive influence on the growth of plants, and in the near future they could be used for increased agricultural production.

## Discussion

Endophytic diazotrophs have tremendous potential applications in agricultural practices because their ability to fix nitrogen can be put to good use [6]. However, more research is needed on bacterial-plant interactions to find out the limiting factors of the relationship. The premise to solve these problems is to isolate and identify the bacteria colonization in plants.

In this research, about 300bp fragments of *nifH* gene were obtained by using PCR from each isolate (Fig 1a), this result further confirmed that we had isolated and obtained some endophytic diazotrophs. Phylogenetic analysis results show that some of the isolates belonged to *Enterobacter* rather than conventional sense endophytic diazotrophs, obviously it was opposite to the study results previously, so we boldly speculate that a number of bacteria which belong to the *Enterobacter* have obtained the nitrogen fixation ability through lateral gene transfer of *nifH*. Lateral gene transfer is important to prokaryote genome evolution and has important practical effects, however, the evolutionary forces of lateral gene transfer is not well known [30]. 879F-RAPD fingerprinting which based on the whole genome of isolates were found to be a magnetic method for the classification and identification of endophytic diazotrophs, and it had an advantage over 16S rRNA RFLP. The study reveals that there are a variety of endophytic diazotrophs in the seeds and stem of crops, however, some strains such as *Enterobacter*, have the ability of nitrogen fixing. Plant assays results suggested that endophytic diazotrophs have potential promoting effects on the growth of plants and could be used in agricultural production.

## Abbreviations

RAPD: randomly amplified polymorphic DNA
RFLP: restriction fragment length polymorphism
